# MicroRNA-19b-3p suppresses gastric cancer development by negatively regulating neuropilin-1

**DOI:** 10.1186/s12935-020-01257-0

**Published:** 2020-05-25

**Authors:** Yingfeng Wei, Sheng Guo, Jianhua Tang, Jianjun Wen, Huifen Wang, Xiaobo Hu, Qiuping Gu

**Affiliations:** 1 Department of Gastroenterology, Ganzhou People’s Hospital, Ganzhou, Jiangxi 341000 China; 2 Department of Liver Diseases, The Fifth People’s Hospital of Ganzhou, Ganzhou, Jiangxi 341000 China; 3 Department of Infectious Diseases, The First Affiliated Hospital of Zhengzhou University, Zhengzhou, 450052 China

**Keywords:** Gastric cancer, NRP1, miR-19b-3p

## Abstract

**Background:**

Gastric cancer (GC) remains one of the most common digestive malignancies worldwide and ranked third causes of cancer-related death. Mounting evidence has revealed that miRNAs exert critical regulatory roles in GC development.

**Methods:**

Immunohistochemistry (IHC) and western blot assay were performed to determine the protein expression levels of neuropilin-1 (NRP1) and mRNA levels were confirmed by quantitative RT-PCR (qRT-PCR) in GC tissues. Kaplan–Meier analysis was performed to evaluate the prognostic value of NRP1 in GC. Knockdown of NRP1 was conducted to analyse its function in vitro and *vivo.* Luciferase reporter assay, western blot and qRT-qPCR were employed to identify the miRNAs which directly targeted NRP1. Furthermore, Bioinformatics analysis and experimental verification were used to explore the potential molecular mechanism and signalling pathway.

**Results:**

In the current study, we revealed that NRP1 was highly expressed in GC tumor tissues and was associated with poor prognosis in GC patients. NRP1 knockdown inhibited GC cell growth, migration and invasion in vitro, while suppressed GC xenograft tumor development in vivo. Bioinformatics analysis predicted that miR-19b-3p down-regulated NRP1 expression by targeting its 3′-UTR. Functional assay demonstrated that miR-19b-3p inhibited GC cell growth, migration and invasion via negatively regulating NRP1. Overexpression NRP1 partially reversed the regulatory effect of miR-19b-3p. Moreover, we showed that miR-19b-3p/NRP1 axis regulated the epithelial-to-mesenchymal transition and focal adhesion in GC, which might contribute the GC development and progression.

**Conclusions:**

Taken together, our findings suggest a regulatory network of miR-19b-3p/NRP1 in GC development. The miR-19b-3p/NRP1 axis might be further explored as a potential diagnostic and therapeutic target in GC.

## Background

Gastric cancer (GC) remains one of the most common digestive malignancies worldwide and ranked third causes of cancer-related death [1]. Technical advances in the past decades greatly improve the diagnosis and treatment of GC, however, the prognosis remains poor as most GC patients are diagnosed at relative advanced stages [2, 3]. The lack of initial symptoms in GC patients and acquired resistant to anti-cancer drugs results in the low 5-year overall survival rate [4]. Thus, the identification of early diagnostic biomarkers and therapeutic targets are of great importance and might further the understanding of GC tumorigenesis and progression.

Neuropilin-1 (NRP1) is a trans-membrane glycoprotein that function as a co-receptor for multiple extracellular ligands such as semaphorins, hepatic growth factor, FGF and TGF-β [5, 6]. Mounting evidence has showed that NRP1 exert a critical role in tumorigenesis and metastasis [7, 8]. In liver cirrhosis, NRP1 promoted angiogenesis via upregulation of VEGFR2 through PI3K/AKT signaling pathway [9]. Targeting NRP1, together with EGFR, attenuated the resistance to EGFR-targeted antibody treatment in non-small cell lung cancer [10]. Specifically, in GC cells, epidermal growth factor could enhance the expression of NRP1 and VEGF [11]. NRP1 expression has been demonstrated to be associated with clinicopathology of GC and promoted GC cell proliferation and migration [12]. NRP1 Inhibition using anti-NRP1 monoclonal antibody suppressed cell migration and invasion via dephosphorylating AKT in GC [13]. In the current study, we explored the expression profile and functional role of NRP1 in GC.

MicroRNAs (miRNAs) are small non-coding RNAs participating in various biological processes including tumorigenesis [14–16]. MiRNAs exert their functions via post-transcriptionally regulating target gene expression [17, 18]. Multiple miRNAs has been discovered to be dysregulated in GC, function as tumor suppressors or oncogenes [19, 20]. For instance, miR-28-5p suppressed AKT phosphorylation and inhibited cell migration and invasion of GC cells while miR-181a acted as an oncogene in GC via negatively regulating Caprin-1 [21, 22]. Meta-analysis suggests that miRNAs could serve as promising biomarkers and have important diagnostic value in GC [20, 23]. However, the dysregulated miRNA profile and its regulatory network in GC are still not fully understood.

In the current study, we found that NRP1 expression was upregulated in GC patients and was associated with poor prognosis. Knockdown of NRP1 inhibited GC cell growth, migration and invasion in vivo, and suppressed xenograft tumor development in vivo. Bioinformatics analysis demonstrated that miR-19b-3p specifically dampened NRP1 expression by binding to its 3′-UTR. MiR-19b-3p exerted its tumor suppressor function via negatively regulating NRP1 and EMT/focal adhesion process. Taken together, our findings reveal a novel regulatory network of miR-19b-3p/NRP1 in GC, which might provide novel diagnostic and therapeutic targets in GC.

## Materials and methods

### Patient specimen

102 paired GC tissues and adjacent nontumorous tissues were collected from patients undergoing surgery at Ganzhou People’s Hospital and the First Affiliated Hospital of Zhengzhou University (GZPH GC cohort) between year 2010 and 2018. Tissue microarray (TMA) were constructed with GC tissue specimen. All patients signed the written informed consent. The Ethics Review Committee of Ganzhou People’s Hospital approved the study.

### Cell culture

GC cell lines (SGC-7901, AGS, MGC-823, MKN-45, BGC-823) and control gastric epithelial cell line GES-1 were purchased from the Cell Bank, Chinese Academy of Science (Shanghai, China). HEK293 cells were from ATCC and maintained in the lab. Cells were cultured in Dulbecco’s Modified Eagle’s Medium (Gibco, USA) supplemented with 10% fetal bovine serum (Clark, USA), 100 U/ml Penicillin, and 100 μg/ml Streptomycin at 37 °C under 5% CO2 in a humidified incubator.

### Cell transfection

SGC-7901 or MGC-823 cells were transiently transfected with si-NRP1 or negative control (NC), miR-19b-3p mimics or NC, miR-19b-3p inhibitor or NC, or pcDNA3.1-NRP1 using lipofectamine 3000 (Invitrogen, USA). SGC-7901 cells were transduced with sh-NRP1 or NC and stably NRP1 knockdown cells were selected using neomycin before cell implantation.

### Real time-quantitative PCR

Complementary DNA was prepared from total RNA using First strand cDNA synthesis Kit (Roche, Germany). NRP1 and miR-19b-3p expression were analyzed by quantitative PCR using SYBR Green master mix (Bio-Rad, USA). GAPDH and U6 were used as control. The primers used in the study were listed as following: NRP1, 5′-ACCCAAGTGAAAAATGCGAATG -3′ and 5′-CCTCCAAATCGAAGTGAGGGTT-3′; miR-19b-3p, 5′-AACAGAAGTTTTGCAGGTTTGCATC-3′ and 5′-CAGTGCAGGGTCCGAGGT-3′; U6, 5′-CCAGUUUACCUAACGCAAUTT-3′ and 5′-TTCACGAATTTGCGTGTCAT-3′; GAPDH, 5′-CTGGGCTACACTGAGCACC-3′ and 5′-AAGTGGTCGTTGAGGGCAATG-3′.

### Western blot

Western blot was performed as previously described [24]. The primary antibodies used in the study were listed as following: NRP1 (Abcam, ab81321), E-cadherin (Abcam, ab194982), vimentin (Abcam, ab92547), N-cadherin (Abcam, ab18203), BMP4 (Abcam, ab200796), ICAM1 (Abcam, ab221777), VCAM1 (Abcam, ab134047), GAPDH (Abcam, ab181602).

### Cell growth assays

Cell growth was analyzed by CCK-8 assay, EdU staining assay and Colony formation assay as previously described [25].

### Transwell assay

Cell invasion was evaluated with transwell chamber (Corning, USA). Briefly, 1 × 10 [5] transfected SGC-7901 or MGC-823 cells in serum-free medium were added to the Matrigel-coated top chamber. The bottom chamber was added with 500 μL DMEM medium containing 10% FBS. After 48 h, the invaded cells were fixed and stained with crystal violet.

### Wound healing assay

SGC-7901 or MGC-823 cells were cultured to form monolayer in 6-well plate. An artificial wound was created using a sterile 200 μL pipette tip. Floating cells were washed awat with PBS and rest cells were cultured in serum-free medium for 48 h. The wound was recorded at 0 h and 48 h to calculate the migration distance.

### Luciferase reporter assay

Luciferase vector containing wild type or mutated 3′-UTR sequence of NRP1 was cloned using pGL3-luc vector (Promega, USA). HEK393 cells were seeded into 24-well plates and transfected with luciferase vector, together with miR-19b-3p mimics or negative control. Relative luciferase activity was analyzed using the Dual-Glo luciferase reporter assay kit (Promega, USA) 48 h later.

### Immunohistochemical (IHC) staining

Briefly, 5 μm paraffin tumor section slides were de-paraffined and blocked with normal rat serum, and then incubated with primary antibodies overnight at 4 °C. Then, slides were incubated with HRP conjugated secondary antibodies, and the specific protein expression was detected using a DAB kit (Vector Lab, USA). The primary antibodies used in the study were listed as following: Ki-67 (Proteintech, 27309-1-AP), NRP1 (Abcam, ab81321), E-cadherin (Abcam, ab194982), vimentin (Abcam, ab92547), N-cadherin (Abcam, ab18203), BMP4 (Abcam, ab200796), ICAM1 (Abcam, ab221777), VCAM1 (Abcam, ab134047).

### Xenograft tumor model

BALB/C nude mice (5–6 weeks, 6 mice/group) were obtained from Vital River Laboratory (Beijing, China). 3 × 10^6^ stable transfected SGC-7901 cells with luciferase reporter were subcutaneously inoculated into the nude mice. Tumor growth was monitored every week and tumor bioluminescence was photographed using an IVIS Lumina II system (Caliper Life Sciences, USA) following the manual. Tumor volume was calculated (length × width^2^/2). Tumor were extracted and weighed at 5 weeks. The animal experiment was approved by the Institutional Animal Care and Use Committee of Ganzhou People’s Hospital.

### Statistical analysis

Results were displayed as mean ± SD and analyzed with GraphPad Prism V6 (Prism, USA). Student *t* test and one-way ANOVA was conducted to calculate the difference between two or more groups. A *p < 0.05 is considered to be statistically significant.

## Results

### NRP1 is highly expressed in GC and is associated with poor prognosis

**Fig. 1:**
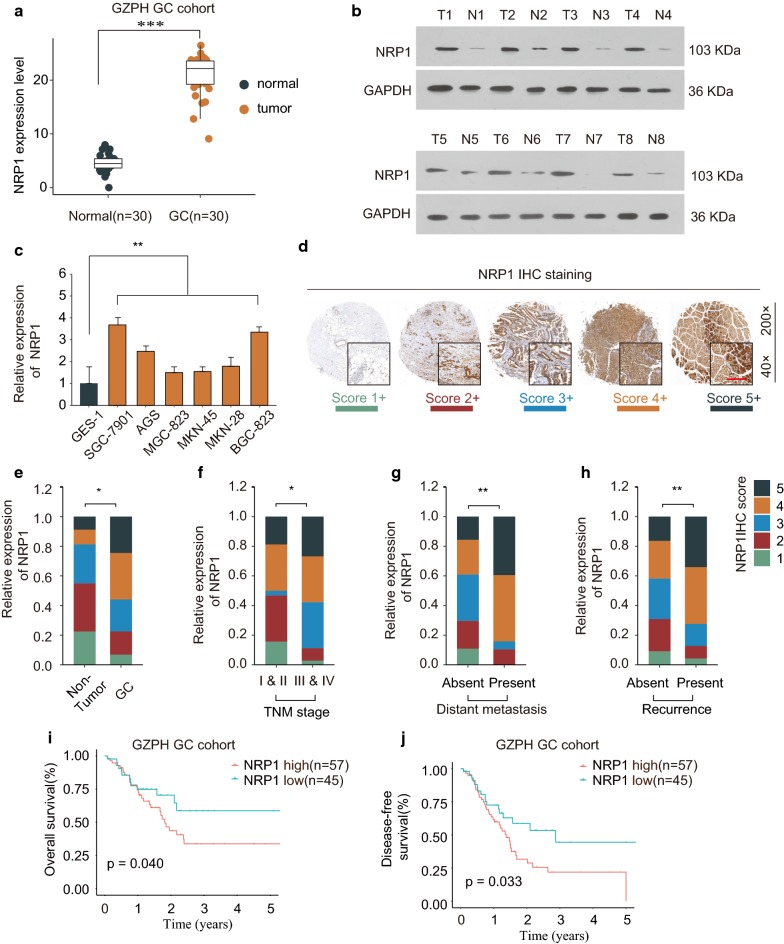
NRP1 is highly expressed in GC and associated with poor prognosis. **a** The mRNA expression of NRP1 in 30-paired GC tissues and adjacent normal tissues from GZPH GC cohort was analyzed by qPCR. **b** The protein expression of NRP1 in 8-paired GC tissues (T) and adjacent normal tissues (N) was analyzed by western blot. **c** The mRNA expression of NRP1 in GC cell lines (SGC-7901, AGS, MGC-823, MKN-45, MKN-28 and BGC-823) and control cell line GES-1 was analyzed by qPCR. **d** IHC staining of NRP1 was performed using GC TMA. The representative NRP1 staining with different staining intensity scores was shown. Scale bars, 200 μm. **e** The distribution of NRP1 IHC staining scores in GC tissues and non-tumor control tissues. (**f–h**) The distribution of NRP1 IHC staining scores in GC with TNM stages I and II or stages III and IV (**f**), with absent or present of distant metastasis (**g**), or with absent or present of recurrence (**h**). **i** Kaplan–Meier analysis of the association between OS and GC patients with high- or low- expression of NRP1. **j** Kaplan–Meier analysis of the association between DFS and GC patients with high- or low- expression of NRP1. *p < 0.05; **p < 0.01; ***p < 0.001

**Fig. 2:**
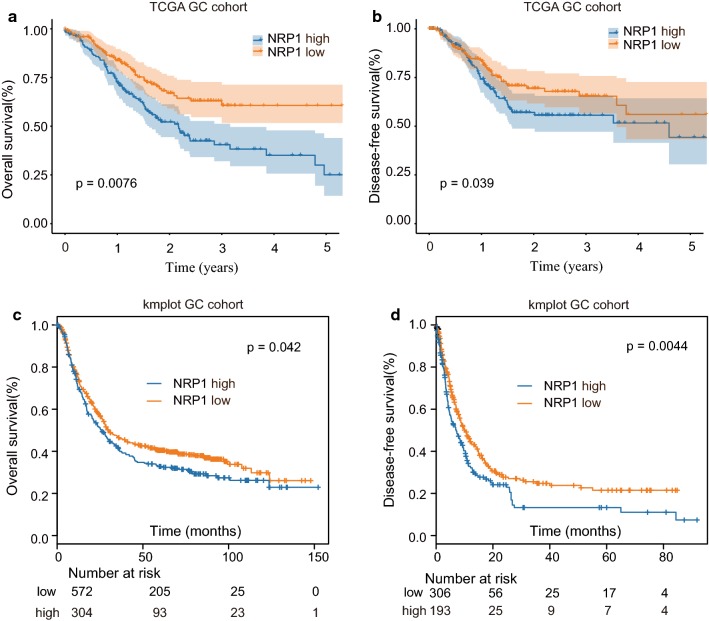
High NRP1 expression is associated with poor prognosis in TCGA GC cohort and kmplot GC cohort. **a**, **b** Kaplan–Meier analysis of the association between OS (**a**) or DFS (**b**) and GC patients with high- or low- expression of NRP1 in TCGA GC cohort. **c**, **d** Kaplan–Meier analysis of the association between OS (**c**) or DFS (**d**) and GC patients with high- or low- expression of NRP1 in kmplot GC cohort

To explore the expression of NRP1, we examined the NRP1 expression in 30-paired GC and adjacent nontumorous tissues. The mRNA levels of NRP1 were significantly upregulated in GC tissues (Fig. 1a). In addition, GC tissues had higher protein expression levels of NRP1 than that in noncancerous normal tissues (Fig. 1b). We further revealed that the expression of NRP1 was notably enhanced in GC cell lines than that in control cell line GES-1 (Fig. 1c). NRP1 IHC staining was performed using GC TMA (Fig. 1d) and GC tissues showed higher NRP1 IHC staining intensity (Fig. 1e). Intriguingly, higher NRP1 expression was significantly correlated with late TNM stages, distant metastasis and recurrence (Fig. 1f–h). Kaplan–Meier analysis showed that patient with high expression of NRP1 had poor overall survival (OS) and disease-free survival (DFS) compared with that in patients with low NRP1 expression in GZPH GC cohort (Fig. 1i–j). We also performed Kaplan–Meier analysis based on TCGA GC cohort and kmplot GC cohort. The results suggested that high NRP1 expression was associated with poor prognosis in both TCGA GC cohort and kmplot GC cohort (Fig. 2a–d). These findings demonstrated that NRP1 expression was upregulated in GC and was associated with poor prognosis.

### Knockdown of NRP1 inhibits GC cell growth, migration and invasion in vitro

**Fig. 3:**
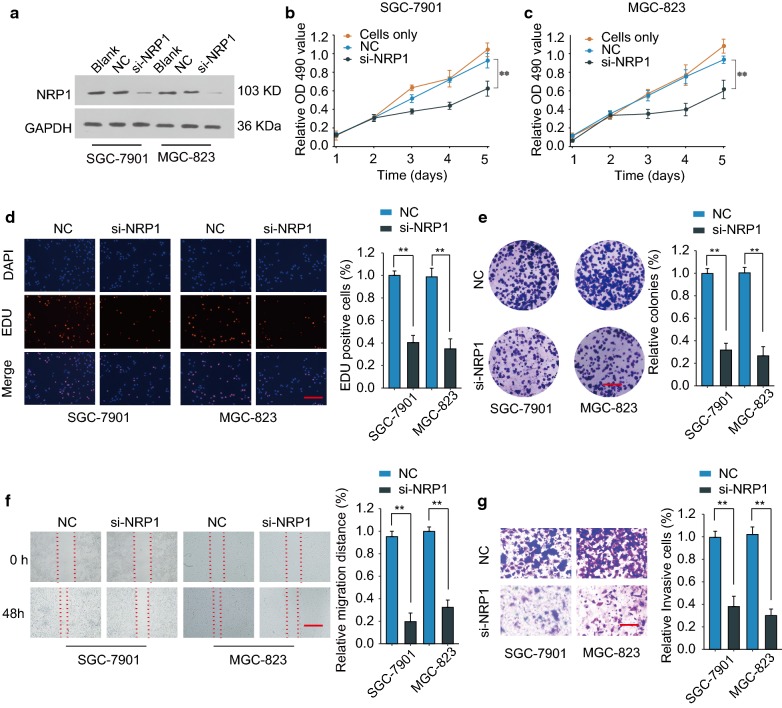
Knockdown of NRP1 inhibits GC cell growth, migration and invasion in vitro. SGC-7901 or MGC-823 cells were transfected with si-NRP1 or negative control (NC). **a** The protein expression of NRP1 was examined by western blot 48 h later. GAPDH was used as an internal loading control. **b**, **c** Cell proliferation was assessed by CCK-8 assay at indicated time points. **d** DNA synthesis was evaluated by EDU/DAPI staining. Scale bars, 50 μm. **e** Cell growth was assessed by colony formation assay. Scale bars, 8 mm. **f** Cell migration was assessed by wound-healing assay. Scale bars, 500 μm. **g** Cell invasion was assessed by transwell assay. Scale bars, 50 μm. *p < 0.05; **p < 0.01

To illustrate the function of NRP1 in GC, RNA silencing was performed to knockdown the expression of NRP1 in GC cells by using siRNA (Fig. 3a). Functionally, knockdown of NRP1 inhibited cell growth, DNA synthesis and colony formation of SGC-7901 and MGC-823 GC cells (Fig. 3b–e). In addition, inhibition of NRP1 suppressed GC cell migration and invasion, as demonstrated by wound-healing assay and transwell assay (Fig. 3f–g). Thus, knockdown of NRP1 inhibits the malignant phenotype of GC cells in vitro.

### Knockdown of NRP1 inhibits GC development and progression in vivo

**Fig. 4:**
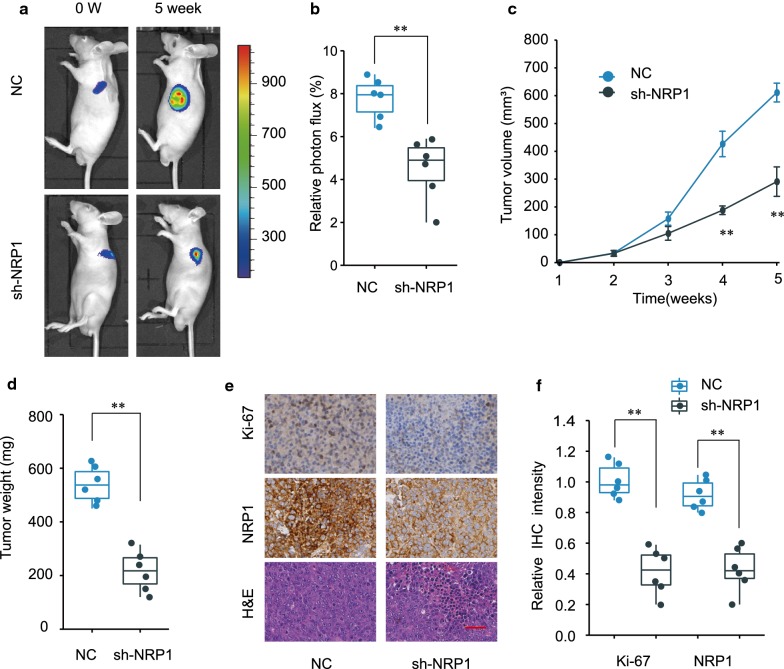
Knockdown of NRP1 inhibits GC development and progression in vivo. SGC-7901 cells were stably transfected with sh-NRP1 or negative control (NC) and subcutaneously inoculated into nude mice. **a** The representative image of nude mice inoculated with SGC-7901 cells was shown at week 0 and week 5. **b** The relative photon flux of GC tumor in nude mice of NC or sh-NRP1 group was analyzed by a live imaging system to measure the luciferase signal. **c** Tumor growth was monitored every week and tumor volume was calculated. **d** The weight of tumors from NC or sh-NRP1 group was analyzed. **e** The representative H&E staining and IHC staining of Ki-67/NRP1 of tumor tissue sections from NC or sh-NRP1 group were shown. Scale bars, 200 μm. **f** The relative IHC staining intensity of Ki-67 and NRP1 in tumor sections from NC or sh-NRP1 group was analyzed. *p < 0.05; **p < 0.01

We further investigated the role of NRP1 in xenograft GC tumor model. SGC-7901 cells stably knockdown NRP1 or control cells were subcutaneously inoculated into nude mice to establish the xenograft tumor. As shown in Fig. 4a–b, knockdown of NRP1 significantly repressed GC tumor growth in vivo. Knockdown of NRP1 markedly slowed tumor growth (Fig. 4c), with a much lower tumor weight (Fig. 4d) compared with control group. Moreover, IHC staining showed that knockdown of NRP1 notably down-regulated the expression of NRP1 and proliferation marker Ki-67 in tumor tissues (Fig. 4e–f). In summary, knockdown of NRP1 suppresses GC tumor development and progression in vivo.

### NRP1 is negatively regulated by miR-19b-3p

**Fig. 5:**
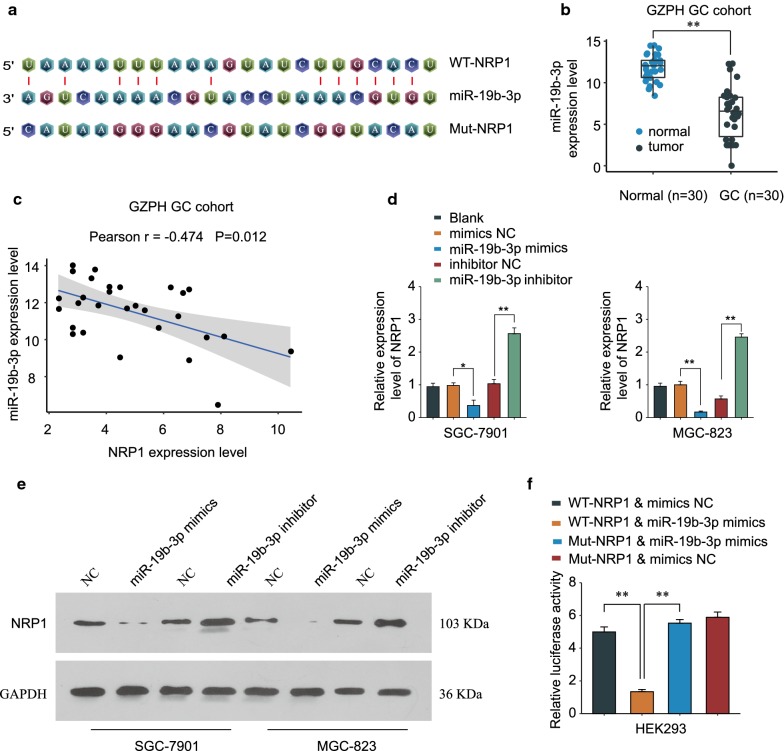
NRP1 is negatively regulated by miR-19b-3p. **a** The putative binding sequences between miR-19b-3p and 3′-UTR of WT NRP1 was predicted by online database miRBase. **b** The expression of miR-19b-3p in GC tissues and adjacent normal tissues of GZPH GC cohort was analyzed by qPCR. **c** Pearson analysis of the association between the expression of miR-19b-3p and NRP1 in GZPH GC cohort. **d**, **e** SGC-7901 or MGC-823 cells were transfected with mimics NC, miR-19b-3p mimics, inhibitor NC or miR-19b-3p inhibitor. The mRNA (**d**) and protein (**e**) expression of NRP1 was examined by qPCR and western blot after 48 h. (**f**) HEK293 cells were transfected with miR-19b-3p mimics or negative control, together with luciferase reporter vector containing WT 3′-UTR of NRP1 (WT-NRP1) or mutated 3′-UTR of NRP1 (Mut-NRP1). The relative luciferase activity was analyzed 48 h later. **p < 0.01

To further understand how NRP1 is regulated in GC development, bioinformatics analysis was performed using online database miRBase to predict the potential miRNAs interacting with NRP1. MiR-19b-3p had the putative binding sequences targeting 3′-URT of WT NRP1 (Fig. 5a). In addition, miR-19b-3p expression was down-regulated in GC tissues in comparison with that in adjacent normal tissues in GZPH GC cohort (Fig. 5b). Pearson correlation analysis revealed that miR-19b-3p expression was negatively associated with NRP1 expression (r = −0.474, P = 0.012, Fig. 5c). Overexpression of miR-19b-3p significantly dampened NRP1 expression, while inhibition of miR-19b-3p enhanced NRP1 expression in SGC-7901 or MGC-823 cells (Fig. 5d–e). Luciferase reporter assay was carried out to further validate the regulation between miR-19b-3p and NPR1. MiR-19b-3p mimics specifically decreased the luciferase activity in HEK293 cells transfected with reporter vector containing WT 3′-UTR of NRP1, but not in cells transfected with reporter vector with mutated 3′-UTR of NRP1 (Fig. 5f). Taken together, the data suggest that NRP1 is negatively regulated by miR-19b-3p.

### MiR-19b-3p regulates GC cell growth, migration and invasion via targeting NRP1

**Fig. 6:**
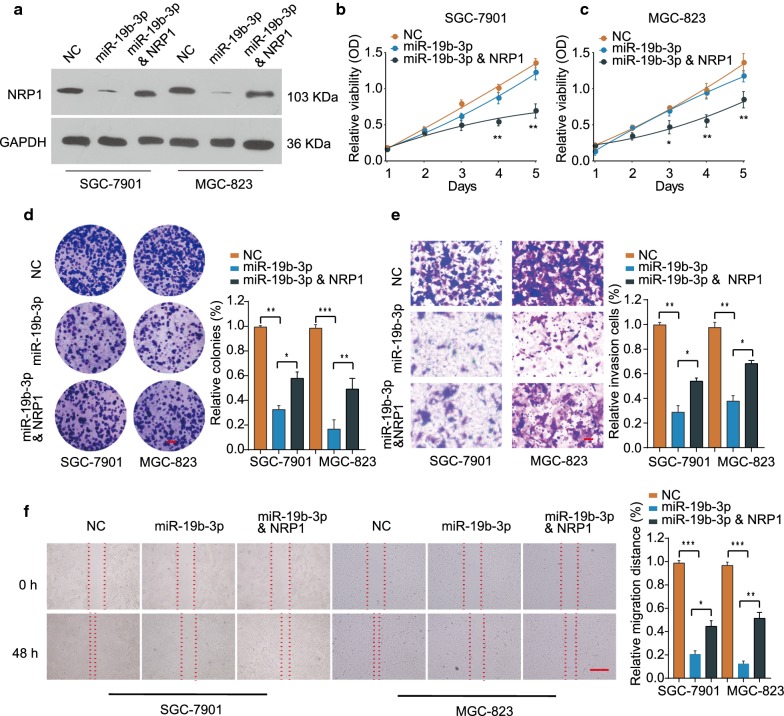
MiR-19b-3p regulates GC cell growth, migration and invasion via targeting NRP1. SGC-7901 or MGC-823 cells were transfected with negative control (NC), miR-19b-3p mimics, or miR-19b-3p mimics + pcDNA3.1-NRP1. **a** The expression of NRP1 was analyzed 48 h later. GAPDH was used as an internal loading control. **b**, **c** Cell proliferation of SGC-7901 or MGC-823 cells was assessed by CCK-8 assay at indicated time points. **d** Cell growth was analyzed by colony formation assay. Scale bars, 8 mm. **e** Cell invasion was analyzed by transwell assay. Scale bars, 50 μm. **f** Cell migration was analyzed by wound-healing assay. Scale bars, 500 μm. *p < 0.05; **p < 0.01

Rescue experiment was conducted to further verify the regulation of NRP1 by miR-19b-3p. Overexpression of miR-19b-3p notably suppressed NRP1 expression while the protein expression of NRP1 was partially restored by NRP1 overexpression in SGC-7901 or MGC-823 cells (Fig. 6a). Functionally, NRP1 overexpression dampened the inhibitory effect of cell growth mediated by miR-19b-3p mimics (Fig. 6b–d). Furthermore, overexpression of miR-19b-3p inhibited GC cell invasion and migration, while the inhibition was reversed by NRP1 overexpression (Fig. 6e–f). These results suggest that miR-19b-3p regulates GC cell growth, migration and invasion via negatively regulating NRP1.

### MiR-19b-3p/NRP1 axis regulates epithelial-mesenchymal transition and focal adhesion

**Fig. 7:**
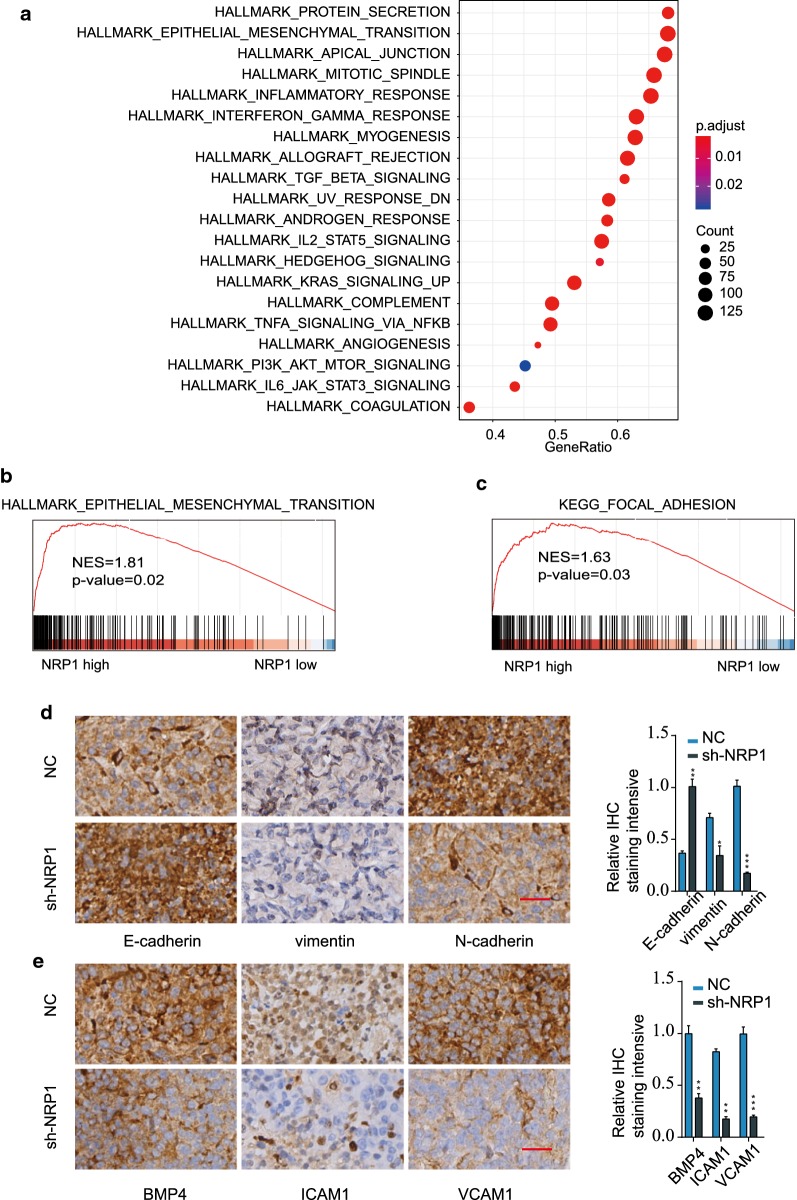
MiR-19b-3p/NRP1 axis regulates epithelial-mesenchymal transition and focal adhesion in GC. **a** GSVA analysis the different regulated signaling pathways in GC tumor compared with adjacent non-tumor tissues. **b**, **c** GSEA analysis the enrichment of signature genes in NRP1 high or low group. **d** IHC staining of E-cadherin, vimentin, and N-cadherin in xenograft tumor tissues from NC or sh-NRP1 group. Scale bars, 200 μm. **e** IHC staining of BMP4, ICAM1, and VCAM1 in xenograft tumor tissues from NC or sh-NRP1 group. *p < 0.05; **p < 0.01

**Fig. 8:**
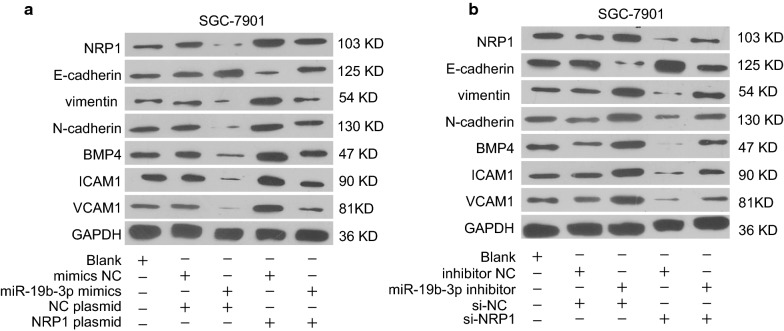
MiR-19b-3p/NRP1 axis regulates epithelial-mesenchymal transition and focal adhesion in GC cells. **a** SGC-7901 cells were transfected with mimic negative control, miR-19b-3p mimics, pcDNA-3.1-NRP1 overexpression vector or negative control pcDNA3.1 vector. The protein expression of NRP1, E-cadherin, vimentin, N-cadherin, BMP4, ICAM1, and VCAM1 was analyzed by western blot 48 h later. **b** SGC-7901 cells were transfected with inhibitor negative control, miR-19b-3p inhibitor, si-NRP1 or negative control si-NC. The protein expression of NRP1, E-cadherin, vimentin, N-cadherin, BMP4, ICAM1, and VCAM1 was analyzed by western blot 48 h later. GAPDH was used as an internal loading control

Gene set variation analysis (GSVA) using TCGA GC datasets was performed to further understand the functional mechanism of NPR1 in GC development. As shown in Fig. 7a, epithelial-mesenchymal-transition (EMT) was among the most upregualted pathways in GC tissues with high NRP1 expression compared with that in GC tissues with low NRP1 expression. Gene Set Enrichment Analysis (GSEA) further validated that EMT and focal adhesion were significantly enriched in NRP1 high group (Fig. 7b–c). IHC staining was performed in xenograft and the results found that knockdown of NRP1 significantly elevated E-cadherin expression and repressed the expression of Vimentin and N-cadherin (Fig. 7d). Moreover, IHC staining revealed that knockdown of NRP1 inhibited the expression of focal adhesion/migration related proteins such as BMP4, ICAM1 and VCAM1 (Fig. 7e). The regulation of EMT and focal adhesion by miR-19b-3p/NRP1 was further tested in SGC-7901 cells. Overexpression of miR-19b-3p or knockdown of NRP1 significantly enhanced E-cadherin expression and suppressed Vimentin/N-cadherin expression, while repressed BMP4, ICAM1 and VCAM1 expression in GC cell lines. The functional effect of miR-19b-3p overexpression was partially reversed by NRP1 overexpression (Fig. 8a). In contrast, miR-19b-3p inhibition promoted EMT-related Vimentin and N-cadherin expression and enhanced focal adhesion with higher expression of BMP4, ICAM1, and VCAM1. Knockdown of NRP1 abrogated the regulatory effect of miR-19b-3p inhibition (Fig. 8b).

## Discussion

Accumulating studies suggest that miRNA/mRNA loop exerts critical function in various human diseases including cancers [26]. In this study, we revealed that oncogene NRP1 was highly expressed in GC and promoted GC development and progression. In addition, we identified that NRP1 was a direct target of miR-19a-3p and miR-19a-3p/NRP1 loop regulated epithelial-to-mesenchymal transition and focal adhesion in GC. Thus, our findings suggest a potential diagnostic and therapeutic target for GC treatment.

GC can be classified into different subtypes according to the different anatomic locations, development stages, or major morphologic components [27]. Based on histology, researchers divided GC into two types: gland formation (GF) and no gland formation (nGF) [28]. Intriguingly, the NPR-1 expression was not significantly different in these two types. However, NRP1 expression was not an independent prognostic factor in the GF group while NRP1 expression was an independent prognostic factor in nGF group and predicted a poor prognosis [28]. Zhuo et al. demonstrated that NRP1 was associated with survival time in patients with advanced GC [29]. In addition, it has been demonstrated that tumor levels of NRP1 might aid the selection of patients with advanced or metastatic GC who might benefit from the combination treatment of bevacizumab and chemotherapy [30]. Our findings further validated the higher expression of NRP1 in GC with advanced TNM stages, distant metastasis, and the presence of recurrence. We also showed that high expression of NRP1 was associated with poor prognosis in GC patients. Taken together, NRP1 is predicted to be a good biomarker and therapeutic target for advanced or metastatic GC treatment.

Studies have shown that NRP1 was regulated by miRNAs in different tumors [31, 32]. MiR-148b was reported to inhibiting NRP1 and regulating cancer stem cells in hepatocellular carcinoma [32]. Hang C et al. recently demonstrated that miR-9 inhibited cell growth and migration via targeting NRP1 in GC [33]. We performed bioinformatics analysis and predicted miR-19b-3p might negatively regulate NRP1 expression via binding to its 3′-UTR. Luciferase reporter assay confirmed the interaction between miR-19b-3p and NPR1. Furthermore, NRP1 expression was negatively associated with miR-19b-3p expression in GC tissues. Studies have demonstrated that there are multiple-to-multiple relationships between miRNAs and target genes in GC [34]. Thus, it is possible that NRP1 was regulated by multiple miRNAs including miR-19b-3p and miR-9 in GC. Further studies will be conducted to address the combined regulatory effect of multiple miRNAs targeting NRP1.

MiR-19b-3p has been investigated in various cancers including breast cancer, pancreatic cancer, colon cancer and so on [35–37]. MicroRNA profiling in patients with GC identified miR-19b-3p was significantly downregulated in GC [38]. Consistently, we found that miR-19b-3p was low-expressed in GC tissues and cel lines. It has been reported that circulating miR-19b-3p was a novel potential biomarker to indicate the progression of GC [39]. However, how miR-19b-3p regulates GC development and progression is not clear. To our knowledge, this is the first report to demonstrate that miR-19b-3p negatively regulates NRP1 expression in GC. Rescue experiments validated that overexpression NRP1 partially abrogated the tumor suppressive function of miR-19b-3p. Consistent with our data, Weiming Chu et al. showed that NRP1 promoted EMT and predicated poor prognosis in human oral squamous cell carcinoma [40]. Intriguingly, anti-NRP1 monoclonal antibody treatment repressed adhesion of MCF7 breast cancer cells [41]. We also demonstrated that miR-19b-3p/NRP1 axis regulated EMT and focal adhesion in GC.

## Conclusion

In summary, our findings verified miR-19b-3p/NRP1 axis plays a critical role in the development of GC. MiR-19b-3p inhibited GC cell growth, migration and invasion via negatively regulating NRP1 and EMT/cell adhesion process. Thus, the results suggest a novel potential diagnostic and therapeutic value of miR-19b-3p/NRP1 in GC.

## Data Availability

All data generated during this study are included in this article.

## Abbreviations

GC: Gastric cancer
NRP1: Neuropilin-1
TMA: Tissue microarray
RT-qPCR: Real-time reverse transcription polymerase chain reaction
IHC: Immunohistochemical
OS: Overall survival
DFS: Disease-free survival

## References

[1] Bray F, Ferlay J, Soerjomataram I, Siegel RL, Torre LA, Jemal A (2018). Global cancer statistics 2018: GLOBOCAN estimates of incidence and mortality worldwide for 36 cancers in 185 countries. CA Cancer J Clin.

[2] Sitarz R, Skierucha M, Mielko J, Offerhaus GJA, Maciejewski R, Polkowski WP (2018). Gastric cancer: epidemiology, prevention, classification, and treatment. Cancer Manag Res.

[3] Yu X, Hu F, Li C, Yao Q, Zhang H, Xue Y (2018). Clinicopathologic characteristics and prognosis of proximal and distal gastric cancer. Onco Targets Ther.

[4] Allemani C, Matsuda T, Di Carlo V, Harewood R, Matz M, Niksic M (2018). Global surveillance of trends in cancer survival 2000–2014 (CONCORD-3): analysis of individual records for 37 513 025 patients diagnosed with one of 18 cancers from 322 population-based registries in 71 countries. Lancet.

[5] Guo HF, Vander Kooi CW (2015). Neuropilin functions as an essential cell surface receptor. J Biol Chem.

[6] Yang ZG, Wen RT, Qi K, Li J, Zheng GX, Wang YF (2019). The Neuropilin-1 ligand, Sema3A, acts as a tumor suppressor in the pathogenesis of acute leukemia. Anat Rec.

[7] Rizzolio S, Cagnoni G, Battistini C, Bonelli S, Isella C, Van Ginderachter JA (2018). Neuropilin-1 upregulation elicits adaptive resistance to oncogene-targeted therapies. J Clin Invest.

[8] Jubb AM, Sa SM, Ratti N, Strickland LA, Schmidt M, Callahan CA (2012). Neuropilin-2 expression in cancer. Histopathology.

[9] Wang L, Feng Y, Xie X, Wu H, Su XN, Qi J (2019). Neuropilin-1 aggravates liver cirrhosis by promoting angiogenesis via VEGFR2-dependent PI3K/Akt pathway in hepatic sinusoidal endothelial cells. EBioMedicine.

[10] Kim YJ, Baek DS, Lee S, Park D, Kang HN, Cho BC (2019). Dual-targeting of EGFR and Neuropilin-1 attenuates resistance to EGFR-targeted antibody therapy in KRAS-mutant non-small cell lung cancer. Cancer Lett.

[11] Akagi M, Kawaguchi M, Liu W, McCarty MF, Takeda A, Fan F (2003). Induction of neuropilin-1 and vascular endothelial growth factor by epidermal growth factor in human gastric cancer cells. Br J Cancer.

[12] Li L, Jiang X, Zhang Q, Dong X, Gao Y, He Y (2016). Neuropilin-1 is associated with clinicopathology of gastric cancer and contributes to cell proliferation and migration as multifunctional co-receptors. J Exp Clin Cancer Res.

[13] Ding Y, Zhou J, Wang S, Li Y, Mi Y, Gao S (2018). Anti-neuropilin-1 monoclonal antibody suppresses the migration and invasion of human gastric cancer cells via Akt dephosphorylation. Exp Ther Med.

[14] Hu M, Zhu S, Xiong S, Xue X, Zhou X (2019). MicroRNAs and the PTEN/PI3K/Akt pathway in gastric cancer (Review). Oncol Rep.

[15] Tan W, Liu B, Qu S, Liang G, Luo W, Gong C (2018). MicroRNAs and cancer: key paradigms in molecular therapy. Oncol Lett.

[16] Peng Y, Croce CM (2016). The role of MicroRNAs in human cancer. Signal Transduct Target Ther.

[17] Vasudevan S (2012). Posttranscriptional upregulation by microRNAs. Wiley Interdiscip Rev RNA.

[18] O’Hara SP, Mott JL, Splinter PL, Gores GJ, LaRusso NF (2009). MicroRNAs: key modulators of posttranscriptional gene expression. Gastroenterology.

[19] Hu ML, Xiong SW, Zhu SX, Xue XX, Zhou XD (2019). MicroRNAs in gastric cancer: from bench to bedside. Neoplasma.

[20] Wei H, Pu K, Liu XG, Li BX, Zhang HS, Wang H (2019). The diagnostic value of circulating microRNAs as a biomarker for gastric cancer: a metaanalysis. Oncol Rep.

[21] Xiao F, Cheng Z, Wang P, Gong B, Huang H, Xing Y (2018). MicroRNA-28-5p inhibits the migration and invasion of gastric cancer cells by suppressing AKT phosphorylation. Oncol Lett.

[22] Lu Q, Chen Y, Sun D, Wang S, Ding K, Liu M (2018). MicroRNA-181a functions as an oncogene in gastric cancer by targeting caprin-1. Front Pharmacol.

[23] Pereira AL, Magalhaes L, Moreira FC, Reis-das-Merces L, Vidal AF, Ribeiro-Dos-Santos AM (2019). Epigenetic field cancerization in gastric cancer: microRNAs as promising biomarkers. J Cancer.

[24] Xue C, He Y, Zhu W, Chen X, Yu Y, Hu Q (2018). Low expression of LACTB promotes tumor progression and predicts poor prognosis in hepatocellular carcinoma. Am JTrans Res.

[25] Bao J, Yu Y, Chen J, He Y, Chen X, Ren Z, Xue C, Liu L, Hu Q, Li J, Cui G (2018). MiR-126 negatively regulates PLK-4 to impact the development of hepatocellular carcinoma via ATR/CHEK1 pathway. Cell Death Dis.

[26] Zhang G, Pian C, Chen Z, Zhang J, Xu M, Zhang L (2018). Identification of cancer-related miRNA-lncRNA biomarkers using a basic miRNA-lncRNA network. PLoS ONE.

[27] Hu B, El Hajj N, Sittler S, Lammert N, Barnes R, Meloni-Ehrig A (2012). Gastric cancer: classification, histology and application of molecular pathology. J Gastrointest Oncol.

[28] Seo HS, Hyeon J, Song IH, Lee HH (2020). Relationship between neuropilin-1 expression and prognosis, according to gastric cancer histology. J Mol Histol.

[29] Zhuo YJ, Shi Y, Wu T (2019). NRP-1 and KDR polymorphisms are associated with survival time in patients with advanced gastric cancer. Oncol Lett.

[30] Van Cutsem E, de Haas S, Kang YK, Ohtsu A, Tebbutt NC, Ming XuJ (2012). Bevacizumab in combination with chemotherapy as first-line therapy in advanced gastric cancer: a biomarker evaluation from the AVAGAST randomized phase III trial. J Clin Oncol.

[31] Zhang G, Chen L, Khan AA, Li B, Gu B, Lin F (2018). miRNA-124-3p/neuropilin-1(NRP-1) axis plays an important role in mediating glioblastoma growth and angiogenesis. Int J Cancer.

[32] Liu Q, Xu Y, Wei S, Gao W, Chen L, Zhou T et al. miRNA-148b suppresses hepatic cancer stem cell by targeting neuropilin-1. Biosci Rep 2015, **35**(4). 10.1042/BSR20150084 PMC4613672 25997710

[33] Hang C, Yan HS, Gong C, Gao H, Mao QH, Zhu JX (2019). MicroRNA-9 inhibits gastric cancer cell proliferation and migration by targeting neuropilin-1. Exp Ther Med.

[34] Hashimoto Y, Akiyama Y, Yuasa Y (2013). Multiple-to-multiple relationships between microRNAs and target genes in gastric cancer. PLoS ONE.

[35] Song M, Sun M, Xia L, Chen W, Yang C (2019). miR-19b-3p promotes human pancreatic cancer Capan-2 cells proliferation by targeting phosphatase and tension homolog. Ann Transl Med.

[36] Jin J, Sun Z, Yang F, Tang L, Chen W, Guan X (2018). miR-19b-3p inhibits breast cancer cell proliferation and reverses saracatinib-resistance by regulating PI3K/Akt pathway. Arch Biochem Biophys.

[37] Jiang T, Ye L, Han Z, Liu Y, Yang Y, Peng Z (2017). miR-19b-3p promotes colon cancer proliferation and oxaliplatin-based chemoresistance by targeting SMAD4: validation by bioinformatics and experimental analyses. J Exp Clin Cancer Res.

[38] Zhang T, Liu C, Huang S, Ma Y, Fang J, Chen Y (2017). A downmodulated MicroRNA profiling in patients with gastric cancer. Gastroenterol Res Pract.

[39] Zhang J, Song Y, Zhang C, Zhi X, Fu H, Ma Y (2015). Circulating MiR-16-5p and MiR-19b-3p as two novel potential biomarkers to indicate progression of gastric cancer. Theranostics.

[40] Chu W, Song X, Yang X, Ma L, Zhu J, He M (2014). Neuropilin-1 promotes epithelial-to-mesenchymal transition by stimulating nuclear factor-kappa B and is associated with poor prognosis in human oral squamous cell carcinoma. PLoS ONE.

[41] Zeng F, Luo F, Lv S, Zhang H, Cao C, Chen X (2014). A monoclonal antibody targeting neuropilin-1 inhibits adhesion of MCF7 breast cancer cells to fibronectin by suppressing the FAK/p130cas signaling pathway. Anticancer Drugs.

